# Structural and Functional Brain Alterations in Post-traumatic Headache Attributed to Mild Traumatic Brain Injury: A Narrative Review

**DOI:** 10.3389/fneur.2019.00615

**Published:** 2019-06-14

**Authors:** Todd J. Schwedt

**Affiliations:** Department of Neurology, Mayo Clinic, Scottsdale, AZ, United States

**Keywords:** post-traumatic headache, traumatic brain injury, concussion, brain imaging, migraine, functional magnetic resonance imaging, diffusion tensor imaging, functional connectivity

## Abstract

**Introduction:** By definition, post-traumatic headache (PTH) attributed to mild traumatic brain injury (mTBI) is not associated with brain structural abnormalities that are seen on routine clinical inspection of brain images. However, subtle brain structural abnormalities, as well as functional abnormalities, detected via research imaging techniques yield insights into the pathophysiology of PTH. The objective of this manuscript is to summarize published findings regarding research imaging of the brain in PTH attributed to mTBI.

**Methods:**For this narrative review, PubMed was searched using the terms “post-traumatic headache” or “post-concussion headache” and “imaging” or “magnetic resonance imaging” or “research imaging” or “positron emission tomography”. Articles were chosen for inclusion based on their relevance to the topic.

**Results:** Ten articles were ultimately included within this review. The studies investigated white matter tract integrity and functional connectivity in acute PTH, structural measures, white matter tract integrity, cerebral blood flow, and functional connectivity in persistent PTH (PPTH), and proton spectroscopy in both acute and persistent PTH. The articles demonstrate that acute and persistent PTH are associated with abnormalities in brain structure, that acute and persistent PTH are also associated with abnormalities in brain function, that it might be possible to predict the persistence of PTH using brain imaging findings, and that there are differences in imaging findings when comparing PTH to healthy controls and when comparing PTH to migraine. Although it is not entirely clear if the imaging findings are directly attributable to PTH as opposed to the underlying TBI or other post-TBI symptoms, correlations between the imaging findings with headache frequency and headache resolution suggest a true relationship between the imaging findings and PTH.

**Conclusions:** PTH attributed to mTBI is associated with abnormalities in brain structure and function that can be detected via research imaging. Additional studies are needed to determine the specificity of the findings for PTH, to differentiate findings attributed to PTH from those attributed to the underlying TBI and coexistent post-TBI symptoms, and to determine the accuracy of imaging findings for predicting the development of PPTH.

## Introduction

Post-traumatic headache (PTH) attributed to traumatic injury to the head is defined by the International Classification of Headache Disorders 3 (ICHD-3) as a headache that is reported to have developed within 7 days of the head injury, regaining consciousness following the head injury, or discontinuation of medication(s) impairing the ability to sense or report headache following the head injury (1). PTH can be a new headache that develops after the injury or it can be significant worsening of a pre-existing headache, such as when there is substantial worsening of migraine patterns following the injury. PTH is considered “acute” when it has been present for 3 months or less, and “persistent” when it has been present for more than 3 months. According to the ICHD-3, PTH can be attributed to mild traumatic head (brain) injury or to moderate or severe traumatic head (brain) injury. An essential component of the ICHD-3 definition for mild traumatic brain injury (mTBI) is that there is no imaging evidence of a traumatic head injury such as skull fracture, intracranial hemorrhage, and/or brain contusion. Thus, by this definition, brain images from individuals with mTBI are clinically interpreted as “normal.” However, as discussed within this article, advanced imaging techniques and imaging data analyses reveal abnormalities in brain structure and function in individuals with PTH attributed to mTBI.

PTH is a highly prevalent symptom following TBI; likely, it is the most common acute and persistent symptom following mTBI (2). Although PTH may be the only symptom during the acute and persistent phases following TBI, often PTH is accompanied by cognitive, mood, sleep, and autonomic symptoms (3, 4). The phenotype of PTH most commonly resembles that of migraine, due to the presence of moderate to severe headache and sensory hypersensitivities, or tension-type headache, although less commonly it resembles other primary headaches (5, 6). Given the phenotypic overlap with primary headaches, like migraine, it is presumed that there are shared underlying mechanisms between PTH and the primary headaches.

Given the high incidence of PTH following TBI, the significant impact that PTH has on individuals, and the absence of PTH-specific therapies, it is essential that we continue to investigate mechanisms for acute PTH and PTH persistence. Research neuroimaging of brain structure and function is one useful method by which to conduct such investigations. In this manuscript, published findings from research neuroimaging studies of acute and persistent PTH are summarized and interpreted, and future directions are discussed.

## Methods

For this narrative review, articles were identified by searching PubMed using the terms “post-traumatic headache” or “post-concussion headache” and “imaging” or “magnetic resonance imaging” or “research imaging” or “positron emission tomography.” The resulting list was reviewed by the author and abstracts and full manuscripts were further reviewed based on the author's judgment of the relevance of each article to the topic. The reference lists of each selected article were reviewed to identify additional publications not identified via the PubMed search. Ultimately, 10 articles were included in this Review.

## Imaging Acute PTH (Table 1)

Diffusion tensor imaging (DTI) allows for assessment of white matter integrity. Alhilali and colleagues performed a DTI analysis of 58 individuals who had PTH of a migraine phenotype attributed to mTBI vs. 17 individuals who had mTBI without PTH of a migraine phenotype (although they could have PTH of a non-migraine phenotype) (7). The median time from injury to evaluation was 20 days. The cohort with PTH of a migraine phenotype had lower fractional anisotropy (FA) in the corpus callosum and fornix/septohippocampal circuit. Furthermore, FA within regions of the fornix/septohippocampal circuit correlated positively with visual memory performance (*r* = 0.325, *p* = 0.01). The corpus callosum and the fornix/septohippocampal circuit have previously been implicated in migraine or cortical spreading depression pathophysiology (10, 11). A major strength of this study is the use of a mTBI group without PTH of a migraine phenotype as a comparator group, theoretically controlling for potential effects of the mTBI on white matter tract integrity. However, it is not clear what proportion of this control group had PTH of a phenotype other than migraine and how the presence of such headaches could impact DTI findings. Furthermore, the correlation between DTI measures in the fornix/septohippocampal circuit with visual memory performance could suggest that the DTI finding is not (entirely) related to the presence of PTH of a migraine phenotype.

**Table 1:** Imaging findings during acute post-traumatic headache.

**References**	**PTH phase**	**Imaging modality or sequence**	**Comparison group(s)**	**Main findings**
Alhilali et al. (7)	Acute	DTI	mTBI with PTH of migraine phenotype vs. mTBI without PTH of migraine phenotype	PTH of migraine phenotype: lower FA in corpus callosum and fornix/septohippocampal circuit.
Delic et al. (8)	Acute	DTI	1) mTBI with PTH of migraine phenotype vs. mTBI without PTH of migraine phenotype 2) mTBI with PTH of migraine phenotype vs. migraine controls and vs. HC	mTBI with PTH of migraine phenotype vs. all other groups: lower SE of FA dataInverse correlation between SE and time to recovery.Accuracy for differentiating PTH of migraine phenotype from a) controls: specificity 95%, sensitivity 77% b) mTBI without PTH of a migraine phenotype: specificity 75%, sensitivity 81%
Ghodadra et al. (9)	Acute	DTI	mTBI with PTH vs. mTBI without PTH	PTH: lower FA in splenium of corpus callosum; higher FA in genu of corpus callosum

*Diffusion tensor imaging studies demonstrate anomalies in white matter tract integrity in acute PTH due to mild traumatic brain injury*.

*DTI, diffusion tensor imaging; mTBI, mild traumatic brain injury; PTH, post-traumatic headache; FA, fractional anisotropy; HC, healthy control subject; SE, Shannon Entropy*.

A principal components analysis of DTI FA data was performed to identify white matter injury patterns associated with PTH attributed to mTBI (9). Sixty-four patients with mTBI were included, 40 with PTH and 24 without PTH. Median duration between injury and presentation was between 3 and 4 weeks. A principal component that included decreased FA in the splenium and increased FA in the genu of the corpus callosum was associated with an increased risk of PTH (odds ratio 2.32, 95% confidence interval 1.29–4.67, *p* = 0.01). This principal component accurately classified those who had PTH with an area under the curve of 0.73.

Delic and colleagues investigated the performance of a classifier based on Shannon entropy (SE) that used FA DTI data in differentiating individuals who had PTH of a migraine phenotype attributed to mTBI from those who had mTBI without PTH of a migraine phenotype and from healthy controls (8). SE measures the complexity of a dataset—the more information or complexity within a dataset, the higher the SE. In the realm of white matter integrity, higher SE of FA data could be reflective of a mixed pattern of white matter tract injury, swelling, and remyelination. It has been hypothesized that SE provides a more accurate measurement of axonal changes that occur after neurologic injury. For this analysis, 57 participants with mTBI and PTH of a migraine phenotype, 17 with mTBI without PTH of a migraine phenotype, 22 healthy control subjects, and 20 control subjects with migraine were included. All mTBI patients had acute PTH with a median time from injury to clinical presentation of 20 days and with 83% of patients presenting within 2 months of injury. Those with mTBI were found to have lower SE compared to controls and there was an inverse correlation between SE and time to patient recovery. PTH of a migraine phenotype was associated with lower SE compared to mTBI without PTH of a migraine phenotype. SE measures provided high accuracy for differentiating those with mTBI from controls (Area under the curve = 0.92, specificity 95%, sensitivity 77%) and for differentiating mTBI with PTH of a migraine phenotype from mTBI without PTH of a migraine phenotype (Area under the curve = 0.85, specificity 75%, sensitivity 81%). This study suggests that SE analyses of DTI data may be particularly useful for investigating white matter pathology and repair following mTBI and for building classification models (i.e., diagnostic biomarkers) for PTH.

## Combined Study of Acute and Persistent PTH Using MR Spectroscopy (Table 2)

MRI proton spectroscopy (MRS) was used to compare N-acetylspartate (NAA, an indicator of neuronal vitality) and choline (a marker of membrane turnover) ratios with creatinine in 17 individuals with PTH attributed to mTBI vs. 12 healthy controls (13). Nine individuals had acute PTH while the remaining 8 had PPTH. Compared to controls, PTH was associated with reduced NAA/creatinine in the anterior regions of the frontal lobe white matter, anterior and posterior medial regions of the frontal lobes, and medial regions of the parietal lobes. Choline/creatinine was increased in regions of the posterior frontal lobe white matter, anterior medial frontal lobe, and medial parietal lobes. Authors concluded that decreased NAA is suggestive of reduced neuronal vitality, likely due to the underlying head trauma, while the increased choline is a marker of membrane turnover and cell proliferation developing around the injured neurons.

**Table 2:** Imaging studies that investigated acute and persistent post-traumatic headache or prediction of persistent post-traumatic headache.

**References**	**PTH phase**	**Imaging modality or sequence**	**Comparison group(s)**	**Main findings**
Obermann et al. (12)^*^	1) Acute 2) Persistent	Gray matter density	1) Acute PTH vs. HC 2) PTH-acute vs. persistent	Acute PTH vs. HC: no differences PPTH vs. HC: less gray matter density in anterior cingulate cortex and dorsolateral prefrontal cortex in PPTH 12 months: those who had PPTH at 3 months, but resolved prior to 12 months had normal gray matter density
Sarmento et al. (13)	1) Acute 2) Persistent	MRS	HC	Reduced NAA/creatinine in frontal lobes (anterior, anterior and posterior medial), parietal lobes (medial). Increased choline/creatinine in frontal lobes (posterior, anterior medial), parietal lobes (medial).
Niu et al. (14)	Acute: predicting development of PPTH	Functional connectivity	PPTH vs. PTH resolution during acute phase	Connectivity of PAG with inferior parietal lobule and with precuneus predicted persistence of PTH: 100% sensitivity and 78% specificity

*A study of gray matter density in those with PTH attributed to whiplash showed less gray matter in PPTH, but not acute PTH, compared with healthy controls. An MRS study that combined individuals with acute PTH with those who had PPTH into one group, found evidence for reduced neuronal vitality, increased membrane turnover and cell proliferation in PTH. A functional connectivity study demonstrated promise for using measures of periaqueductal gray connectivity for predicting the persistence of PTH*.

*PTH, post-traumatic headache; HC, healthy control subject; MRS, magnetic resonance spectroscopy; NAA, N-acetylspartate; PPTH, persistent post-traumatic headache; PAG, periaqueductal gray*.

* *PTH attributed to whiplash*.

## Predicting the Persistence of PTH Using Brain Imaging

Fortunately, many individuals with acute PTH have headache resolution during the first few days to weeks following PTH onset and thus do not have persistence of PTH (2). However, up to 60% do not have PTH resolution, their PTH endures for more than 3 months, and they are classified as having PPTH (2). The ability to predict who will have persistence of PTH could be valuable for several reasons: (1) those at high risk for PPTH might benefit from closer clinical follow-up; (2) early intervention might reduce the impact of PTH and might prevent PTH persistence (although specific interventions for preventing PTH persistence have yet to be identified); and (3) those individuals at high risk for PPTH would be appropriate for clinical trials of therapeutics aimed at treating acute PTH and reducing the risk for PTH persistence.

An MRI resting-state functional connectivity study by Niu and colleagues suggests that prediction of PPTH is possible (14). In this study, 54 patients with mTBI and acute PTH had brain MRIs including functional connectivity measurements within 7 days of their injury, and they had a 3-month follow-up evaluation to assess for PTH persistence. Investigators analyzed functional connectivity with the right ventrolateral periaqueductal gray (PAG), since this region has been shown to have abnormal connectivity in other pain studies, since it is vulnerable to mTBI, and since it plays a role in opioid antinociception (15–17). At baseline, compared to healthy controls, those with acute PTH had weaker right ventrolateral PAG functional connectivity with several regions of the default mode network, including regions in the right and left precuneus, right inferior parietal lobule, and right angular gyrus, and stronger connectivity with a region in the left middle temporal gyrus. There were significant negative correlations between PAG with right precuneus connectivity and Headache Impact Test 6 scores and between PAG with right inferior parietal lobule and Headache Impact Test 6 scores when measured at baseline. Furthermore, baseline connectivity between the PAG with the right inferior parietal lobule and with the right precuneus were predictors for having persistence of PTH measured at 3 months. A logistic regression model including age, sex, years of education, loss of consciousness duration, and functional connectivity of the PAG with the default mode network predicted PPTH with 100% sensitivity (95% confidence interval (CI) 66%−100%) and 78% specificity (95% CI 63%−89%). This study demonstrates that acute PTH is associated with altered functional connectivity of the PAG with regions of the default mode network, that the connectivity strength correlates with PTH burden, and that functional connectivity measured during the acute post-TBI phase contributes to prediction of PTH persistence.

## Imaging Persistent PTH (Table 3)

An MRI study by Chong and colleagues compared vertex-by-vertex cortical thickness measurements in 33 patients with PPTH due to mTBI with 33 healthy controls (18). Those with PPTH had a median of 7 years with PPTH and 16 days per month with headache and had no history of migraine prior to TBI. After adjustment for several factors that could impact cortical thickness, including age, sex, depression scores, and anxiety scores, the PPTH group had significantly less cortical thickness in left and right frontal (superior frontal, caudal middle frontal, precentral) and right parietal (precuneus, supramarginal, inferior parietal, superior parietal) regions compared to healthy controls. Considering these regions that differed between subject groups, there were significant negative correlations between the thickness of the left and right superior frontal regions with headache frequency. There were no significant correlations between the cortical thickness of these regions and years lived with PPTH. These findings suggest a relationship between the severity of PTH (measured as headache frequency in this case) with brain structure. Relationships between the severity of post-TBI symptoms with measures of brain structure have been suggested in other studies, although not specifically investigating PTH (23, 24). The association between headache frequency with cortical thickness, but not years of PTH with cortical thickness, at least suggests that the finding of less cortical thickness is attributable to PTH as opposed to the underlying mTBI.

**Table 3:** Imaging findings during persistent post-traumatic headache.

**References**	**PTH phase**	**Imaging modality or measure**	**Comparison group(s)**	**Main findings**
Chong et al. (18)	Persistent	Cortical thickness	HC	Less thickness in frontal (superior, caudal middle, precentral) and parietal lobes (precuneus, supramarginal, inferior, superior).
				Negative correlations between superior frontal thickness with headache frequency.
Chong et al. (19)	Persistent	DTI	1) Migraine 2) HC	vs. Migraine: DTI differences in anterior thalamic radiations, cingulum, inferior and superior longitudinal fasciculi, uncinate fasciculi, corticospinal tract.
				Positive correlation between cingulum angular bundle MD and RD with headache frequency.
				vs. HC: DTI differences in anterior thalamic radiations, cingulum, corticospinal tract, inferior longitudinal fasciculus, uncinate fasciculus, forceps major and minor.
Gilkey et al. (20)	Persistent	CBF	1) Migraine 2) HC	vs. Migraine + HC: reduced regional CBF; greater regional and hemispheric CBF asymmetries.
Schwedt et al. (21)	Persistent	Regional volumes, cortical thickness, surface area, curvature	1) Migraine 2) HC	vs. Migraine: structural differences in frontal (lateral orbitofrontal, caudal middle, superior) and parietal lobes (precuneus, supramarginal).
				vs. HC (only considering regions that differed between PPTH vs. Migraine): frontal (lateral orbitofrontal, superior) and parietal lobes (supramarginal).
Dumkrieger et al. (22)	Persistent	Static and dynamic functional connectivity	1) Migraine 2) HC	vs. Migraine (static connectivity): 17 region pairs that included somatosensory, insula, hypothalamus, anterior and middle cingulate, temporal pole, supramarginal, superior parietal, middle occipital, lingual, pulvinar, precuneus, cuneus, somatomotor, ventromedial prefrontal, and dorsolateral prefrontal regions.
				Correlations between years with headache and headache frequency with static connectivity of a few region pairs.
				vs. Migraine (dynamic connectivity): 10 region pairs that included somatosensory, hypothalamus, middle cingulate, temporal pole, supramarginal, superior parietal, lingual gyrus, somatomotor, precentral, posterior cingulate, middle frontal, fusiform, parieto-occipital, and amygdala regions.
				Correlations between headache frequency and pain intensity with dynamic connectivity with a couple region pairs.
				vs. HC (only considering regions that differed between PPTH vs. Migraine; static connectivity): dorsolateral prefrontal, ventromedial prefrontal, middle cingulate, somatomotor, pulvinar, insula, hypothalamus.
				vs. HC (only considering regions that differed between PPTH vs. Migraine; dynamic connectivity): somatosensory, lingual, middle cingulate, supramarginal, temporal pole, middle frontal.

*Studies investigating brain structure and regional cerebral blood flow demonstrate differences between those with PPTH vs. healthy controls and vs. those with migraine. Correlations between some of the structural measures with headache frequency provide additional evidence for a relationship between the structural changes with PPTH*.

*HC, healthy control subject; DTI, diffusion tensor imaging; MD, mean diffusivity; RD, radial diffusivity; CBF, cerebral blood flow; PPTH, persistent post-traumatic headache*.

Studies that compare brain imaging findings in those with PPTH to individuals with other headache types, like migraine, can provide insights into shared and distinct pathophysiology. Regional cerebral blood flow, measured by the xenon-133 inhalation technique, was investigated in 35 individuals with PPTH attributed to mTBI vs. 49 non-headache controls and 92 individuals with migraine (20). Compared to healthy controls and individuals with migraine, those with PPTH had reduced regional cerebral blood flow, and greater regional and hemispheric asymmetries. The authors suggested that cerebral blood flow changes and vasomotor instability may be contributing mechanisms to the development of PTH and other post-TBI symptoms.

An MRI investigation of regional volumes, cortical thickness, surface area, and brain curvature found structural differences between PPTH and migraine cohorts in the right lateral orbitofrontal lobe, left caudal middle frontal lobe, left superior frontal lobe, left precuneus, and right supramarginal gyrus (21). Amongst these regions that differed between PPTH and migraine, the structure of regions in the right lateral orbitofrontal lobe, right supramarginal gyrus, and left superior frontal lobe also differed between PPTH and healthy controls who had no history of head trauma or migraine. A DTI study by Chong and colleagues investigated node-by-node white matter tract integrity in PPTH vs. migraine and vs. healthy controls (19). DTI measures (mean diffusivity or radial diffusivity) differed in PPTH vs. migraine for several white matter tracts including the anterior thalamic radiations, cingulum, inferior longitudinal fasciculi, uncinate fasciculi, corticospinal tract, and superior longitudinal fasciculi. Amongst those with PPTH there was a positive correlation between cingulum angular bundle mean diffusivity and radial diffusivity with headache frequency, a relationship not seen amongst those with migraine. A study by Dumkrieger and colleagues compared static and dynamic functional connectivity of 59 regions of interest involved in pain processing in PPTH vs. migraine (22). Between group differences in static and dynamic functional connectivity were identified for several regions and correlations between connectivity strength and headache burden (i.e., years with headache, headache frequency) were found. These studies demonstrate that despite significant overlap in symptoms between migraine and PPTH (89–96% of those with PPTH in these studies had a phenotype consistent with “migraine” or “probable migraine”), there are identifiable differences in brain structure and function, perhaps suggesting distinct underlying pathophysiology. Further studies are necessary to determine the specificity of the differences for PPTH and to determine if the findings are directly related to PPTH vs. being attributable to the underlying mTBI irrespective of PPTH.

## Limitations and Future Directions

One of the biggest challenges in research imaging of PTH is differentiating imaging findings associated with PTH vs. those attributable to the underlying TBI vs. those associated with other post-TBI symptoms. mTBI is associated with acute, subacute and perhaps even chronic changes in brain structure and function (25). The persistence of post-TBI symptoms is associated with the presence and duration of these imaging abnormalities (26, 27). When symptoms are present, PTH is often just one symptom of many that the individual with mTBI experiences. In addition to PTH, mTBI is often associated with alterations in mood, cognition, sleep patterns, and non-headache pain (28). The presence of brain imaging abnormalities due to the mTBI itself and the common scenario of PTH being one of a constellation of symptoms, makes it challenging to isolate the brain imaging findings associated with PTH from those associated with the mTBI or other symptoms. Research designs that include cohorts who have mTBI without active symptoms and mTBI with symptoms not including headache, as well as longitudinal designs that include imaging before and after resolution of symptoms could help identify imaging findings specifically associated with PTH.

Additional studies are necessary to determine if imaging findings associated with PTH are shared by primary headaches, by other secondary headaches, or by non-headache pain types, or if there are imaging findings that are specific for PTH. This determination would lend insights into the pathophysiology of PTH and could contribute to the objective classification of PTH via models that include brain imaging data.

It is not yet known if the mechanism of brain injury leading to PTH impacts research imaging findings. A study by Obermann and colleagues measured regional gray matter using voxel based morphometry in 32 patients who had PTH attributed to whiplash (12). In this longitudinal study, patients were imaged within 14 days of injury and again after 3 months; those with headaches that lasted longer than 3 months (*n* = 12) were imaged again at 1 year post-injury. When imaged within the first 14 days post-injury, there were no significant regional differences in gray matter density between those with PTH and controls. For those who went on to have PPTH, there were significant decreases in gray matter density in the anterior cingulate cortex and dorsolateral prefrontal cortex when comparing the 14 days to 3 months post-injury scans. The 3-month changes in gray matter density resolved by 1 year, coinciding with headache resolution in all but one patient. The temporal correlation between the structural findings with the presence, persistence and resolution of PTH strongly suggest that the imaging findings are directly related to PTH. Furthermore, although no direct comparisons have been made between PTH attributed to whiplash with PTH attributed to TBI, the anterior cingulate cortex and dorsolateral prefrontal cortex are regions that have been commonly identified as participating in headache physiology as well as non-headache pain physiology (29, 30).

The ability to predict which individuals with acute PTH will have persistence of PTH would be a major advance for the clinical management of individuals with acute PTH and for optimizing enrollment of individuals into clinical trials of PTH therapeutics. Although much work needs to be done to understand the optimal treatments for acute and persistent PTH, presumably, the early identification of the patient who is likely to have persistence of PTH would allow for earlier and more aggressive management of acute PTH with pharmacologic and non-pharmacologic therapies. Earlier therapeutic intervention might be more effective than treatment that is started after PTH has already become persistent and might even prevent the persistence of PTH. Furthermore, to reduce unnecessary risks and to limit required patient sample sizes, it would be optimal if clinical trials of preventive therapies for acute PTH could enroll patients who are at high risk of having PTH persistence, excluding those likely to have natural PTH resolution. A prognostic biomarker for PPTH could assist with appropriate enrollment into such clinical trials.

## Conclusions

Research neuroimaging is starting to provide insights into the pathophysiology of acute and persistent PTH. Further work is needed to determine if there are imaging findings that are specific to PTH, as opposed to being attributable to the underlying trauma or post-injury symptoms that accompany PTH. Determining the specificity of imaging findings for PTH will deepen our understanding of PTH pathophysiology and might contribute to the ability to diagnostically classify new onset PTH from worsening of a pre-injury primary headache. Furthermore, imaging might contribute to models that predict which individuals with acute PTH are likely to develop PPTH, a prediction that would be useful for determining how aggressive to manage those with acute PTH and for determining a cohort of individuals who would be most appropriate for clinical trials of PTH therapeutics.

## Author Contributions

The author confirms being the sole contributor of this work and has approved it for publication.

### Conflict of Interest Statement

The author declares that the research was conducted in the absence of any commercial or financial relationships that could be construed as a potential conflict of interest.
